# Interdisciplinary Assessment of Hygiene Practices in Multiple Locations: Implications for COVID-19 Pandemic Preparedness in Vietnam

**DOI:** 10.3389/fpubh.2020.589183

**Published:** 2021-01-26

**Authors:** Trang Huyen Thi Nguyen, Huong Thi Le, Xuan Thi Thanh Le, Toan Thanh Thi Do, Toan Van Ngo, Hai Thanh Phan, Giang Thu Vu, Tu Huu Nguyen, Dung Tri Phung, Son Hong Nghiem, Thuc Minh Thi Vu, Thu Ha Nguyen, Trung Dinh Tran, Khanh Nam Do, Dat Van Truong, Thanh Tuan Le, Bach Xuan Tran, Carl A. Latkin, Roger C. M. Ho, Cyrus S. H. Ho

**Affiliations:** ^1^Faculty of Medicine, Duy Tan University, Da Nang, Vietnam; ^2^Institute for Global Health Innovations, Duy Tan University, Da Nang, Vietnam; ^3^Institute for Preventive Medicine and Public Health, Hanoi Medical University, Hanoi, Vietnam; ^4^Center of Excellence in Evidence-Based Medicine, Nguyen Tat Thanh University, Ho Chi Minh City, Vietnam; ^5^Vietnam Young Physicians' Association, Hanoi, Vietnam; ^6^School of Medicine, Griffith University, Gold Coast, Parklands Drive, Southport, QLD, Australia; ^7^Centre for Applied Health Economics, Griffith University, Brisbane, QLD, Australia; ^8^Dai Nam University, Hanoi, Vietnam; ^9^Department of Epidemiology, Demography and Medical Statistics, Faculty of Public Health, Danang University of Medical Technology and Pharmacy, Da Nang, Vietnam; ^10^University of Medicine and Pharmacy at Ho Chi Minh, Ho Chi Minh City, Vietnam; ^11^Vietnam National Heart Institute, Bach Mai Hospital, Hanoi, Vietnam; ^12^Bloomberg School of Public Health, Johns Hopkins University, Baltimore, MD, United States; ^13^Department of Psychological Medicine, Yong Loo Lin School of Medicine, National University of Singapore, Singapore, Singapore; ^14^Institute for Health Innovation and Technology, National University of Singapore, Singapore, Singapore; ^15^Department of Psychological Medicine, National University Hospital, Singapore, Singapore

**Keywords:** coronavirus, sanitation practice, pandemic prevention, local preparedness, hygiene practice

## Abstract

Due to the shared border with China, Vietnam faced risks from the COVID-19 pandemic at the early stages of the outbreak. Good hygiene practices were considered an effective prevention method, but there were only minimal data on the effectiveness of hygiene practices against the pandemic at the community level. Thus, this study aims to assess hygiene practices in society by using a community-based survey. A cross-sectional study using survey monkey was conducted from December 2019 to February 2020. The Snowball sampling technique was used to recruit participants and exploratory factor analysis was applied to scrutinize the construct validity of the measurement. We used the Tobit regression model to assess the association. Hygiene in a high-risk environment and hygiene in the social and educational environment were two main factors after applying the EFA method. Participants grade community sanitation quite low (around 6 out of 10). Furthermore, the mean score of hygiene practice at a local level in a high-risk environment was slightly low at 6.0. The score of sanitation in the Central region (5.3) was quite low compared to the North (5.8) and the South (6.2). The most high-risk environment was construction, industrial zone and food safety. Moreover, younger respondents were more likely to report poorer hygiene practices in high-risk environments (Coefficient = −1.67; 95% CI = −3.03; −0.32) and social and educational environment (Coefficient = −1.29; 95% CI = −2.54; −0.04). Our study gives an insight into pandemic preparedness at the grassroots level. The findings suggest the necessity of specific communication education for society to improve the compliance of hygiene practices to prevent the spreading of COVID-19.

## Introduction

Vietnam is a tropical country where many epidemic risks, including SARS in 2002, Avian Influenza A (H5N1) in 2004 (1), and Dengue (2), have occurred. These outbreaks not only affect population health directly but also have an impact on the economy and society. Since January 2020, Vietnam has faced the risk of COVID-19 case-importation from China, where new coronavirus (COVID-19) pandemic was booming (3). This new virus has affected human respiratory systems and caused severe acute respiratory syndrome (3). According to the most updated information, more than two million patients have been affected by COVID-19 globally, and nearly a hundred thousand people have died due to the infected of this virus (4). Europe was the region most affected, with 880,106 confirmed cases, followed by the Americas, with 573,940 confirmed cases reported by the World Health Organization (WHO) as of April 12, 2020 (4).

The previous study recommended that good hygiene practices were a protective way to reduce the spread of infectious diseases in lower-middle-income countries for both health care providers and society (5). Moreover, in order to control infectious disease, personal hygiene, food safety, clean water, and good hygiene practices were mentioned in the book “Guide to Ship Sanitation” (6). In Vietnam, earlier work demonstrated the obstacles of hygiene practice and disease control in the rural area of Vietnam, including lack of inter-sectoral collaboration, lack of information, and unspecific intervention for the ethnic group (7). In “Healthy Villages: A Guide for Communities and Community Health Workers,” WHO indicated that many communicable diseases can be controlled by using good hygiene as a barrier (8). In conformity with WHO suggestions for COVID-19 prevention, the most effective ways to prevent community spread are frequent hands washing, social distancing, and respiratory hygiene (9). Hence, poor hygiene might increase the speed and the rate of the virus spread.

For pandemic preparedness, Fatiregun et al. suggest a framework for pandemic preparedness to respond to the Lassa fever outbreak (10), which involved the participation of the political system, communities, and the general population. The involvement of communities in pandemic preparedness may strengthen the national surveillance system. Currently, international mechanisms that evaluate national responsiveness to health issues mostly focuses on the health care system, for example, the State Party Annual Reporting (SPAR) from International Health Regulation (IHR), WHO (11). However, the empirical data on hygiene practices at the community level are very sparse and not regularly updated.

Moreover, each country has an action plan regarding disease control depending on the health care system, resources, and characteristics of community involvement. In Western countries, they have “gatekeepers” as the primary clinicians to provide health care services and patients' first point of contact (12). Meanwhile, LMICs such as Vietnam have a system of preventive medicine, and at the grassroots level, it has community services, health professionals, and unions (students, professional, women, framers). This specific feature makes it very interesting to analyze the preparedness for disease control in Vietnam. In the past, Vietnam successfully stopped infectious disease and the SARS epidemic in 2002 as well as the Dengue epidemic. These pathogens, as well as the prevention and control of the two infectious diseases they caused, were well-documented (13, 14). The new coronavirus's characteristics are different from those of MERS and SARS previously (15), in their transmission from human body fluids, droplets, and surfaces. In addition, experiences on epidemic preparedness of other countries might not be applicable in the context of Vietnam. Thus, understanding the characteristics of Vietnamese patients and healthcare system is crucial in preparedness and response to epidemics and the COVID-19 pandemic.

Vietnam is a country significantly affected by infectious diseases, especially seasonal infectious diseases. Previous studies have been limited in evaluating the effectiveness of disease prevention methods at the community level. To reduce the knowledge gap, it is necessary to conduct more studies with more subjects to explore and develop effective prevention methods. Therefore, we conducted this study among healthcare providers, medical students and community workers who are the key force in Vietnam in disease prevention. Assessing hygiene practices can give an insight into the urgency of the COVID-19 pandemic and society's preparedness. Thereby, given these findings we can suggest effective prevention within a local setting.

## Methods and Materials

The project, titled “Outbreak Response Assessment,” included healthcare workers, community workers, and other unions (medical students, youth unions) as their three main subjects.

### Study Setting and Participants

#### Study Design

Using survey monkey, we conducted a cross-sectional study from December 2019 to February of 2020. This study is part of the project “Dataset of Collaboration in Disease Control and Prevention Between Health Professionals and Community Workers at the Grass-Roots Level in Vietnam.” The study was conducted in the early period of the global COVID-19 epidemic.

#### Research Population and Inclusion Criteria

We recruited participants with inclusion criteria including (1) age of 18 or above, (2) Vietnamese residency, (3) agreement to participate in the study and signing the informed consent, and (4) being able to answer the survey.

### Sample Size and Sampling Method

The Snowball sampling technique was applied to recruit participants. We first contacted health care workers of the Vietnam Young Physician Association, Vietnam Youth Federation, or community workers in each province or city and sent them the links of online survey through email, then asked them to invite their co-workers and colleagues to involve in our study. Medical participants were students form medical schools in three cities, including Hanoi, Danang and Ho Chi Minh. A total of 7,733 Vietnamese residents participated in this online survey which consisted of a socio-economic characteristics part and 6 different blocks whose data were analyzed for 6 different studies. Block randomization was adopted as a delivery questionnaire method to reduce selection bias. The data of this study were drawn from a block among 6 ones mentioned above. After removing all respondents who had missing answers, there were 601 health care workers, community workers, and medical students were enrolled in our study.

### Measure and Instruments

Data on demographic characteristics, including gender, age, marital status, and living areas were collected.

To evaluate of hygiene practices, we asked participants to assess the level of hygiene practices in their living area in a seven-item questionnaire, including hygiene in raising, transporting, and slaughtering animals; industrial parks and production units; construction; schools; the provision of clean and running water; food safety; and residential areas. The respondent weighs hygiene practices on a scale from 0 to 10, with 0 considered to represent the poorest hygiene.

### Data Analysis

STATA software (Stata Corp. L, College Station, TX) was used to perform all statistical analyses. We applied exploratory factor analysis (EFA) to assess the construct of the measurement, and Cronbach alpha was used to measure the internal consistency of the questionnaire. In factor analysis, the cutoff mean eigenvalue was above 0.5 to select factors. The threshold was defined by the Scree test. We used orthogonal varimax rotation with Kaiser normalization to reorganize items in the scale, which aimed to increase the interpretability of these factors. A value of 0.4 was utilized as a cutoff point for factor loadings. We also performed a cross-loading on one item and then assigned it to the appropriate domain based on both the nature of the question and the overarching dimension. Cronbach alpha was used to assess the internal consistency and reliability of the measurement.

We used frequencies and proportions to describe the qualitative variables. We reported the quantitative variables with a normal distribution by means and standard deviations. Additionally, the Kruskal-Wallis-test investigated the median differences in hygiene practice scores. Statistical significance was indicated by a *p*-value <0.05. The Wald test was used stepwise with *p*-values ≤ 0.2 to include variables in the model. The Tobit regression was used to evaluate the association between the characteristics of the individual and evaluation of hygiene practice.

### Ethical Considerations

The study protocol received approval by the Scientific Committee of the Scientific Council of Central Vietnam Youth Union Committee (37aQD/TWDTN-VNCTN). All participants provided informed consent, and we explained our questionnaire's objectives fully as well as the confidentiality principle of the measurement. Respondents enrolled in our research only after they fully understood and agreed to participate in our survey. All participants could refuse or stop answering the questionnaire at any time without any discrimination.

## Results

The demographic characteristics of the participants are shown in Table 1. A total of 601 respondents participated in our study. The majority of the participants were medical students from the South of Vietnam (89.7%). Urban citizens accounted for 86.6% of participants, and about 95% of the people in the sample were single. Among the total number of respondents, most were under 25 (80.8%) and worked at the College/University level (62.3%).

**Table 1:** Socio-economic characteristics of respondents.

	**Northern**		**Central**		**South**		**Total**	
	***n***	**%**	***n***	**%**	***n***	**%**	***n***	**%**
**Total**	180	30.0	40	6.7	381	63.4	601	100.0
**Objects**								
Medical professionals	22	12.3	8	20.0	14	3.7	44	7.4
Medical students	124	69.3	18	45.0	340	89.7	482	80.6
Community workers	33	18.4	14	35.0	25	6.6	72	12.0
**Gender**								
Male	60	33.3	18	45.0	101	26.6	179	29.8
Female	120	66.7	22	55.0	279	73.4	421	70.2
**Living area**								
Urban	152	84.4	22	55.0	346	91.3	520	86.8
Rural	28	15.6	18	45.0	33	8.7	79	13.2
**Marital status**								
Single	139	77.2	23	57.5	361	95.0	523	87.2
Living with spouse	40	22.2	16	40.0	16	4.2	72	12.0
Others	1	0.6	1	2.5	3	0.8	5	0.8
**Administration level of workplace**								
Center	35	20.1	5	12.8	43	11.5	83	14.2
Province	25	14.4	15	38.5	65	17.4	105	17.9
Below province	14	8.1	4	10.3	15	4.0	33	5.6
College/University	100	57.5	15	38.5	250	67.0	365	62.3
**Participated in community activities**								
Yes	106	58.9	27	67.5	171	45.0	304	50.7
No	74	41.1	13	32.5	209	55.0	296	49.3
**Age group**								
Under 25	115	70.1	19	47.5	320	89.4	454	80.8
25 and above	49	29.9	21	52.5	38	10.6	108	19.2
	**Mean**	**SD**	**Mean**	**SD**	**Mean**	**SD**	**Mean**	**SD**
**Age**	24.4	7.1	27.0	7.3	21.4	3.5	22.7	5.4

Table 2 describes the reliability of hygiene practices at a local level. In our factor analysis, the cutoff mean eigenvalue was above 0.5 to select a factor. Seven items were classified into two main domains, including hygiene in the workplace and hygiene in a social or educational environment. The alpha ranged from 0.91 to 0.93. However, the mean score of hygiene practices at the local level in a high-risk environment and in social and educational environments was slightly low, at 6.0 and 6.5, respectively.

**Table 2:** Local hygiene practices (Band score 0–10).

**Items**	**Total 10 score**		**Hygiene in workplaces environment**	**Hygiene in a social, educational environment**
	***n***	**%**		
Hygiene in schools	83	13.4		0.82
Hygiene in providing clean water and running water	76	12.3		0.90
Hygiene and food safety	67	10.8		0.92
Hygiene in residential areas	64	10.3		0.91
Hygiene in raising, transporting, and slaughtering animals	63	10.2	0.95	
Hygiene in industrial parks and production units	61	9.9	0.93	
Hygiene in construction	50	8.1	0.94	
**Cronbach alpha**			0.93	0.91
**Mean**			6.0	6.5
**SD**			2.2	2.1

The evaluation of hygiene practices in preparedness for the disease is shown in Table 3. The score for hygiene practices was at a moderate level. As we can see, the overall hygiene in both high-risk environments and in social and education environments in the Central region (mean = 5.3; SD = 2.1) was quite low compared with the North (mean = 5.8; SD = 2.3) and the South (mean = 6.2; SD = 2.2). In the North, the mean score for hygiene practices in construction (mean = 5.7; SD = 2.6) and food safety (mean = 5.9; SD = 2.6) were the lowest compared with other items.

**Table 3:** Evaluation of hygiene practice in preparedness for disease responses.

	**Northern**		**Central**		**South**		**Total**		***p*-value**
	**Mean**	**SD**	**Mean**	**SD**	**Mean**	**SD**	**Mean**	**SD**	
**Hygiene in workplace environment**	**5.8**	**2.3**	**5.3**	**2.1**	**6.2**	**2.2**	**6.1**	**2.2**	**0.01**
Hygiene in raising, transporting and slaughtering animals	5.8	2.4	5.2	2.4	6.3	2.3	6.1	2.4	0.01
Hygiene in industrial parks and production units	6.0	2.3	5.6	2.1	6.3	2.3	6.1	2.3	0.11
Hygiene in construction	5.7	2.6	5.2	2.2	6.2	2.3	6.0	2.4	0.01
**Hygiene in social, educational environment**	**6.3**	**2.1**	**5.7**	**1.8**	**6.7**	**2.0**	**6.5**	**2.1**	**<0.01**
Hygiene in schools	6.9	2.0	6.2	2.0	7.2	2.1	7.1	2.1	<0.01
Hygiene in providing clean water and running water	6.4	2.3	6.4	1.9	7.0	2.1	6.7	2.2	0.01
Hygiene and food safety	5.9	2.6	5.1	2.4	6.3	2.5	6.1	2.5	0.01
Hygiene in residential areas	6.0	2.5	5.2	2.2	6.2	2.5	6.1	2.5	0.02

*The bold values indicate the average score of the factor in the group which were calculated by the exploratory factor analysis*.

Table 4 gives the factors associated with community-based evaluation of hygiene practices at the grassroots level. Respondents older than 25 were more likely to report poorer hygiene practices in high-risk environments (Coefficient = −1.67; 95% CI = −3.03; −0.32) and social and educational environments (Coefficient = −1.29; 95% CI = −2.54; −0.04). Compared with single individuals, those who were married were more likely to report better hygiene practices. The regression model was chosen with forward stepwise variable selection. Therefore, the final model suggested a potential association with the outcome, including the subjects' field of work and region.

**Table 4:** Factors associated with evaluating hygiene practices.

	**Hygiene in social, educational environment**		**Hygiene in high risk environment**	
	**Coef.**	**95% CI**	**Coef.**	**95% CI**
**Subject** (vs. Medical professional)				
Medical students	0.34	−0.77; 1.46	0.33	−0.86; 1.52
Community workers	−0.19	−1.14; 0.76	−0.46	−1.34; 0.43
**Marital status** (vs. single)				
Living with spouse	0.89^**^	0.04; 1.75	0.94^**^	0.11; 1.78
Others	−0.71^*^	−1.52; 0.10	−0.98^*^	−1.99; 0.04
**Age group** (25 and above vs. under 25)	−1.66^**^	−2.96; −0.37	−1.28^*^	−2.66; 0.11
**Regions** (South vs. North)			−0.49	−1.11; 0.14

** *p < 0.05*,

* *p < 0.1*.

## Discussion

Our study reflects a comprehensive evaluation of the community with respect to hygiene practices in a local setting. Our findings indicate that very few participants assessed the level of sanitation in their areas at the highest score, which showed that the level of hygiene practices at the local level was quite low. We observed disparities in sanitation practices in each region across the country. People in the Central region present hygiene practices inferior to those of the North and the South. The most important areas to improve hygiene practice were construction areas and industrial zones, followed by food safety. Therefore, what is needed to improve epidemic preparedness is the establishment and enhancement of community education and the identification of the characteristics of each area to take appropriate protective and preventive measures.

The level of sanitation in high-risk environments was lower in the Central region and the North than in the South of Vietnam. The mean hygiene score in the Central region (mean = 5.2; SD = 2.1) was significantly lower than in the South. This finding was consistent with a previous study on sanitation in agriculture in the North-Central region of Vietnam. The authors revealed that most of people in their study sample used latrine waste with their bare hand for rice production (16). However, a recent study among Vietnamese residents has indicated that the perception of the risk of the novel coronavirus was lower among people in the North of Vietnam (17). The authors explained that the result might be due to the preferences of Chinese travelers for the southern regions of Vietnam. Therefore, people in this region might be more aware of the risk of COVID-19 infection. There are even recommendations to compose and process human excreta for at least six months to reduce the risk of infectious diseases, but only a small number of farmers followed that suggestion because they were more concerned about the smell of excreta than the risks involved (16). Sanitation in construction areas and industrial zones also presents a challenge for a responsive disease-control system. Our results show poor hygiene practices in this high-risk environment. Industrial zones and construction areas have plenty of workers. A previous study on the hygiene practices among bridge painters indicated the popularity of non-compliance with occupational safety and hygiene practices at construction sites, and this behavior has led to the high risk of hazardous exposure through the skin, from surfaces, and from vehicles (18). This information reflects the different levels of population concentration, personal protection equipment, and knowledge in each region. Consequently, a locally responsive system can identify high-risk areas. Therefore, although the population's mobility is high, it is more important to consider the ability of people in the high-density population to protect themselves from the virus, such as in industrial parks or hospital cafeterias. Vietnam has performed quite well in the prevention of epidemics when the country has prioritized national safety for a very long period of the first 30 to 40 days and seems to combat the epidemic effectively. However, the reality shows that Vietnam is facing an epidemic stemming from a restaurant attached to a central hospital. This problem started with the assurance that food safety measures would preserve public health in Vietnam. The findings from our study also showed that the lack of hygiene practices with respect to food safety (mean = 6.1; SD = 2.5), which was comparable with the data of SPAR on the capacity of Vietnam to provide a mechanism for food safety, was at 6 out of 10 (19). Consequently, ensuring food safety and hygiene is extremely important, especially in places where diseases are easily transmitted, as in a hospital. After adjusting for possible confounding factors, our model showed that older participants are more likely to evaluate the lower score of hygiene practice. This result can explain that because the younger participants were mostly medical students, they might not be too concerned about local hygiene practices, which is in line with the previous study on hygiene practices among medical students (20). The author concluded that there are gaps in personal hand hygiene and the importance of hand cleaning techniques among medical students (20).

This study has several implications. While the previous study examined only hygiene practices in a specific setting, our study almost alone among very few studies, has examined various locations and reflected the practices of community service workers, health professionals, and medical students. Thus, it provides a relative comparison to identify and prioritize the locations at high risk. The unique characteristics of each region across the country provide a great deal of information on disease control. Hence, we see the need to develop the specific protection and prevention in the Central region, providing more personal protective equipment to enhance sanitation practices for disease prevention. Additionally, our findings pointed out that sanitation practices and disease prevention need to be organized more adequately and depend on each community structure and increase the multi-sectoral coordination at a local setting. Therefore, during an epidemic of localized transmission, hygiene intervention should especially target construction areas, industrial zones, and schools.

We should reveal the limitations of our study. It is not possible to conclude that there is a causal relationship, due to the cross-sectional study design. The snowball sampling technique may lead to selection bias and not generalize the findings to the general population.

In conclusion, our finding reveals insufficient hygiene practices for disease prevention at the grassroots level in preparedness for the prevention of the epidemic. Our results suggest the improvement and strengthening of community education on hygiene and an appropriate and cost-effective way to prevent infectious diseases, especially COVID-19. Moreover, we also express the necessity of identifying each region's characteristics and classifying the groups at high risk in order to develop disease control programs at the grassroots levels.

## Data Availability Statement

The raw data supporting the conclusions of this article will be made available by the authors, without undue reservation.

## Ethics Statement

The studies involving human participants were reviewed and approved by The Scientific Committee of the Scientific Council of Central Vietnam Youth Union Committee (No. 37aQD/TWDTN-VNCTN). The patients/participants provided their written informed consent to participate in this study.

## Author Contributions

THTN: conceptualization, methodology, investigation, writing—original draft, supervision, and validation. BXT: writing—original draft. HTL: writing—review and editing, and supervision. XTTL, TTTD, TVN, TDT, TMTV, THaN, DT, and TTL: writing—review and editing. HTP and KND: formal analysis. HTP: methodology. GTV: conceptualization and project administration. THuN and DVT: validation. DTP and SN: investigation and conceptualization. CL: conceptualization, methodology, investigation, and supervision. CH: conceptualization, methodology, and investigation. RH: writing—review and editing, conceptualization, methodology, and investigation. All authors contributed to the article and approved the submitted version.

## Conflict of Interest

The authors declare that the research was conducted in the absence of any commercial or financial relationships that could be construed as a potential conflict of interest.
