# Corrigendum: Interdisciplinary Assessment of Hygiene Practices in Multiple Locations: Implications for COVID-19 Pandemic Preparedness in Vietnam

**DOI:** 10.3389/fpubh.2022.854543

**Published:** 2022-02-10

**Authors:** Trang Huyen Thi Nguyen, Huong Thi Le, Xuan Thi Thanh Le, Toan Thanh Thi Do, Toan Van Ngo, Hai Thanh Phan, Giang Thu Vu, Tu Huu Nguyen, Dung Tri Phung, Son Hong Nghiem, Thuc Minh Thi Vu, Thu Ha Nguyen, Trung Dinh Tran, Khanh Nam Do, Dat Van Truong, Thanh Tuan Le, Bach Xuan Tran, Carl A. Latkin, Roger C. M. Ho, Cyrus S. H. Ho

**Affiliations:** ^1^Faculty of Medicine, Duy Tan University, Da Nang, Vietnam; ^2^Institute for Global Health Innovations, Duy Tan University, Da Nang, Vietnam; ^3^Institute for Preventive Medicine and Public Health, Hanoi Medical University, Hanoi, Vietnam; ^4^Center of Excellence in Evidence-Based Medicine, Nguyen Tat Thanh University, Ho Chi Minh City, Vietnam; ^5^Vietnam Young Physicians' Association, Hanoi, Vietnam; ^6^School of Medicine, Griffith University, Gold Coast, Parklands Drive, Southport, QLD, Australia; ^7^Centre for Applied Health Economics, Griffith University, Brisbane, QLD, Australia; ^8^Dai Nam University, Hanoi, Vietnam; ^9^Department of Epidemiology, Demography and Medical Statistics, Faculty of Public Health, Danang University of Medical Technology and Pharmacy, Da Nang, Vietnam; ^10^University of Medicine and Pharmacy at Ho Chi Minh, Ho Chi Minh City, Vietnam; ^11^Vietnam National Heart Institute, Bach Mai Hospital, Hanoi, Vietnam; ^12^Bloomberg School of Public Health, Johns Hopkins University, Baltimore, MD, United States; ^13^Department of Psychological Medicine, Yong Loo Lin School of Medicine, National University of Singapore, Singapore, Singapore; ^14^Institute for Health Innovation and Technology, National University of Singapore, Singapore, Singapore; ^15^Department of Psychological Medicine, National University Hospital, Singapore, Singapore

**Keywords:** coronavirus, sanitation practice, pandemic prevention, local preparedness, hygiene practice

In the published article, the affiliations of the author Bach Xuan Tran is not correct. Instead of having affiliations “Institute for Global Health Innovations, Duy Tan University, Da Nang, Vietnam” and “Bloomberg School of Public Health, Johns Hopkins University, Baltimore, MD, USA”, they should have “Institute for Preventive Medicine and Public Health, Hanoi Medical University, Hanoi, Vietnam” and “Bloomberg School of Public Health, Johns Hopkins University, Baltimore, MD, USA”.

The authors apologize for this error and state that this does not change the scientific conclusions of the article in any way. The original article has been updated.

## Publisher's Note

All claims expressed in this article are solely those of the authors and do not necessarily represent those of their affiliated organizations, or those of the publisher, the editors and the reviewers. Any product that may be evaluated in this article, or claim that may be made by its manufacturer, is not guaranteed or endorsed by the publisher.

